# THI Modulation of Genetic and Non-genetic Variance Components for Carcass Traits in Hanwoo Cattle

**DOI:** 10.3389/fgene.2020.576377

**Published:** 2020-12-23

**Authors:** Yoonji Chung, Seung Hwan Lee, Hak-Kyo Lee, Dajeong Lim, Julius van der Werf, S. Hong Lee

**Affiliations:** ^1^Department of Animal Science and Biotechnology, Chungnam National University, Daejeon, South Korea; ^2^Department of Animal Biotechnology, Chonbuk National University, Jeonju, South Korea; ^3^Division of Animal Genomics and Bioinformatics, National Institute of Animal Science, Rural Development Administration, Wanju, South Korea; ^4^School of Environmental and Rural Science, University of New England, Armidale, NSW, Australia; ^5^Australian Centre for Precision Health, University of South Australia, Adelaide, SA, Australia; ^6^UniSA Allied Health and Human Performance, University of South Australia, Adelaide, SA, Australia

**Keywords:** multivariate reaction norm model, genotype by environment (G×E) interaction, temperature-humidity index, Hanwoo cattle, carcass traits

## Abstract

The phenotype of carcass traits in beef cattle are affected by random genetic and non-genetic effects, which both can be modulated by an environmental variable such as Temperature-Humidity Index (THI), a key environmental factor in cattle production. In this study, a multivariate reaction norm model (MRNM) was used to assess if the random genetic and non-genetic (i.e., residual) effects of carcass weight (CW), back fat thickness (BFT), eye muscle area (EMA), and marbling score (MS) were modulated by THI, using 9,318 Hanwoo steers (*N* = 8,964) and cows (*N* = 354) that were genotyped on the Illumina Bovine SNP50 BeadChip (50K). THI was measured based on the period of 15–45 days before slaughter. Both the correlation and the interaction between THI and random genetic and non-genetic effects were accounted for in the model. In the analyses, it was shown that the genetic effects of EMA and the non-genetic effects of CW and MS were significantly modulated by THI. No significant THI modulation of such effects was found for BFT. These results highlight the relevance of THI changes for the genetic and non-genetic variation of CW, EMA, and MS in Hanwoo beef cattle. Importantly, heritability estimates for CW, EMA, and MS from additive models without considering THI interactions were underestimated. Moreover, the significance of interaction can be biased if not properly accounting for the correlation between THI and genetic and non-genetic effects. Thus, we argue that the estimation of genetic parameters should be based on appropriate models to avoid any potential bias of estimates. Our finding should serve as a basis for future studies aiming at revealing genotype by environment interaction in estimation and genomic prediction of breeding values.

## Introduction

Hanwoo is an indigenous beef cattle breed in Korea, originated from a Bos taurus breed in north-east Asia (Kim and Lee, 2000; Yoon et al., 2005; Węglarz, 2010; Lee et al., 2014). The breed has been raised in an environment where the temperature is significantly different across seasons and regions. Hanwoo has been bred over the last four decades using artificial selection to improve production traits such as carcass weight, eye muscle area and marbling score (Seideman et al., 1987; Jo et al., 2012). Recently, the Korea meat trade association (KMTA) reported that the meat of the breed has been exported to beef markets in Hong Kong and Vietnam and has the potential to expand into broader global markets. Hanwoo is characterized by good intramuscular fat (IMF) that influences consumers perception of meat quality due to sensory properties including tenderness, juiciness and flavor (Albrecht et al., 2011; Irie et al., 2011). Compared to other beef breeds such as Australian Angus, having high subcutaneous fat depth, Hanwoo beef shows higher marbling scores and increased IMF contents (Cho et al., 2005). More importantly, Hanwoo produces highly qualified meat with a high omega-3 fatty acid counts and high proportion of mono-unsaturated fatty acids (MUFAs), which are known to improve lipid profile (e.g., reducing low- and increasing high-density lipoprotein) (Jo et al., 2012; Gotoh and Joo, 2016; Joo et al., 2017).

The national breeding system for Hanwoo includes two genetic evaluation programs, individual performance and progeny test, in which candidate bulls are selected based on their own growth performance, and they are further evaluated with a progeny test (Park et al., 2013; Chung et al., 2018). From the breeding program, 30 proven bulls are selected every year and their semen is distributed to farms with different environments across the nation (Park et al., 2013; Chung et al., 2018). Environmental factors can significantly affect the performance of economic traits such as production efficiency, feed intake and weight gain in Hanwoo. Among various environmental factors, thermal effects, e.g., heat stress, have been considered as a significant factor to determine the performance of beef cattle. The genetic variance of economic traits in beef cattle can be significantly changed by thermal effects (Bradford et al., 2016; Santana et al., 2016). Santana et al. (2016) reported that the estimated heritability for weaning weight was significantly different across different levels of temperature-humidity index (THI) in three Brazilian beef cattle breeds, Nellore, Brangus and a tropical composite breed. It was also reported that there were heterogeneous genetic variances across different levels of temperature for weaning weight in Angus cattle (Espasandin et al., 2013; Bradford et al., 2016). In dairy cattle, a number of studies reported that the genetic variance of dairy production traits varied significantly with levels of THI (Aguilar et al., 2009).

To estimate genotype-by-environment (G×E) interaction, two kinds of models have mainly been used in previous studies, i.e., a bivariate linear mixed model for modeling effects of distinct environments and a reaction norm model (RNM) for effects of continuous environmental gradients. Bivariate linear mixed models can detect G×E interaction by estimating a genetic correlation between different environmental groups for the trait of interest and test if the estimated genetic correlation is significantly different from 1, using the Wald test statistics. For instance, Ibi et al. (2005) used this approach and estimated a genetic correlation for carcass traits in Japanese black cattle to be significantly different from 1 between two groups that produced under different temperature and heat stress-related conditions. Another approach that can be followed is to apply an RNM that estimates random regression coefficients of genetic (and non-genetic) effects on a continuous environmental variable and tests whether the variance of the regression slope is significantly different from 0. For example, Santana et al. (2016) estimated a significant variance of the random regression slope for weaning weight on THI in a tropical composite breed of Brazilian cattle, implying that there is genetic variation in how animals cope with THI differences.

While most interaction studies focus on G×E interaction (genetic heterogeneity), it has also been recognized that the heterogeneity of residual variance exists. Residual variance heterogeneity may be due to outliers, scale effects or non-normality of data, which can be remedied by standard quality control including normalization (e.g., rank-based inverse normal or log transformation) (Box and Hill, 1974; Downs and Rocke, 1979; Atkinson et al., 2016). However, residual can include some effects, which are not captured in the model, but have biological functions, such as the effects of gene-expression, methylation or unrecorded environmental factors (Soto-Ramírez et al., 2013; Romanowska et al., 2019). Such residual effects can be modulated by THI, which is referred to as residual-by-environment (R×E) interaction in this study.

In livestock, genetic and residual correlations play a critical role in genetic evaluation. However, most G×E interaction studies have overlooked the fact that correlations between the main trait and an environmental modulator, i.e., genotype-environment (G-E) correlation, can cause spurious G×E interaction signals (Hill, 1984; Ni et al., 2019; Zhou et al., 2020). Although THI is an environmental factor, there can be a non-negligible correlation between THI and the genetic effects on production traits, i.e., G-E correlation (Sellers et al., 1998; Ni et al., 2019; Zhou et al., 2020). It is also possible that there is a significant correlation between THI and the residual variance of production traits, i.e., residual-environment (R-E) correlation (Könyves et al., 2017; Ni et al., 2019; Zhou et al., 2020). However, to date, there are few or no studies to consider correlation and interaction simultaneously in G×E studies in livestock (Supplementary Table 1), and it is not clear how to account for G-E or R-E correlations when estimating the genetic effects of production traits modulated by THI. In this study, we model G×E and R×E interactions jointly for four carcass traits in Hanwoo cattle that may be modulated by THI, using a multivariate reaction norm model (MRNM). In the model, we explicitly estimate correlations between THI and the genetic and residual effects of carcass traits, which prevent spurious interaction signals due to confounding between correlation and interaction.

## Materials and Methods

### Animal and Phenotype Data

A total of 10,215 Hanwoo Korean cattle (9,856 steers and 359 cows) born between 2006 and 2016 were included in this study. The data were provided by the BioGreen 21 Program (Molecular Breeding Program) of National Institute of Animal Science (NIAS), Rural Development Administration (RDA), South Korea, and the experimental procedures were approved by The Animal Care and Use Committee of the NIAS, RDA, South Korea. The contemporary group (CG) was based on the classification of individuals belonging to the same year of birth, season, resulting in 29 contemporary groups. Farm information was collected from 15 provinces located in the northern [Gangwon (# farms is 2,265), Incheon (527), Gyeonggi (20)], middle [Chungbuk (559), Chungnam (553), Daejeon (19), Sejong (176), Gyeongbuk (826)] and southern regions [Gyeongnam (1,732), Deagu (5), Ulsan (261), Jeonbuk (555), Jeonnam (1,031), Gwangju (17), and Jeju (8)] of the Republic of Korea. The average temperature in the southern area is about 3–6°C higher than that of north area.

Phenotypic data in this study included carcass weight (CW), back fat thickness (BFT), eye muscle area (EMA), and marbling score (MS) (Supplementary Table 2). BFT, EMA, and MS were measured at the 12th-13th rib junction after a 24-h chill. MS was recorded on a scale ranging from 1- to 9-grade, which was assessed by trained evaluators of the Korea Institute of Animal Products Quality Evaluation (KAPE). Among 10,215 cattle, only 10,201 animals had more than 5 records in each CG and farm location (province) and were used in the main analysis.

### Meteorological Data

We used the meteorological data from 15 regional weather stations nearest to the farms. Weather variables used in our study included daily maximum temperature and daily average humidity (Lee et al., 2019). THI records were used as an environmental variable for reaction norm model. Following National Research Council [NRC] (1971), the THI can be written as

THI=(1.8×T+32)-[(0.55-0.0055×H)×(1.8×T-26.8)]

where *T* and *H* are the average maximum daily temperature (Celsius scale) and average relative humidity (%), which were measured between 15 and 45 days before the slaughter date (Dikmen and Hansen, 2009). We considered this period because the season at slaughter is reported to be critical for carcass traits, and the last month before the slaughter would be the most important period for the quality of carcass traits (Kadim et al., 2004; Pestana et al., 2012; Hagenmaier et al., 2016). We used the weather information measured between 15 and 45 days before the slaughter date because animals would be facing to the fasting time, pre-slaughter rest period, and the transportation to the slaughterhouse, which might take approximately 15 days (Hagenmaier et al., 2016). The THI values range from 39.60 to 94.06 with a mean of 73.98 for the Hanwoo cattle.

### Genotyping and Quality Control

The DNA data was extracted from Longissimus dorsi muscle samples using a DNeasy Blood Tissue Kit. NanoDrop 1000 (Thermo Fisher Scientific, Wilmington, DE) was used for DNA concentration and purity, and SNP marker data (58,990 SNPs) was obtained using the Illumina Bovine SNP50 BeadChip (50K) platform. Quality control procedures were applied to SNP filtering using PLINK 1.9. software (Purcell et al., 2007). SNPs were excluded if they were on the sex chromosomes, their call rate was less than 0.10 and their minor allele frequency was less than 0.01. Furthermore, those animals that had a significant departure from Hardy-Weinberg equilibrium (<0.0001) and individual missingness more than 0.1 were removed from the analyses (Bolormaa et al., 2011; Bhuiyan et al., 2018), which remained 9,318 animals (8,964 steers and 354 cows). For the phenotypes of each carcass trait, we excluded records outside +/− three standard deviations from the phenotypic mean. After this stringent quality control, a total of 40,118 SNPs remained, and the number of animals with phenotypic records were 9,243, 9,202, 9,241, and 9,317 for CW, BFT, EMA and MS, respectively (Supplementary Table 2).

### Statistical Analyses

In preliminary analyses, to avoid any confounding effects, the phenotypes of each trait were adjusted for fixed effects such as sex, CG, farm location and age. We additionally tested if there were significant linear or quadratic fixed effects of THI on the phenotypes. We also accounted for genetic population structure by fitting the first 10 principal components (PCs) estimated from the genomic relationship matrix. For this, we used eight different linear models, and applied model comparisons using Akaike information criteria (AIC) (Supplementary Table 3). According to the AIC, the phenotypes of CW and EMA needed to be adjusted for both linear and quadratic THI and the first 10 PCs. BFT were adjusted for quadratic THI and the first 10 PCs, whereas MS was only adjusted for the first 10 PCs only. The pre-adjusted phenotypes of each trait from these analyses were standardized and rank-based inverse transformed (RINT), to avoid any violation against the normality assumption of RNM (Supplementary Figure 1). The variance components were estimated using the pre-adjusted RINT phenotypic data in the main analysis (Ni et al., 2019; Shin and Lee, 2020; Zhou et al., 2020).

An association between THI and genetic effects can be revealed by checking whether the genomic relationships between the samples (which were inferred from their genotypes in this study) explains any of the variation in THI. Such an association would then lead to a spurious heritability estimate of THI, obviously not because of THI truly having a heritable component, but because of confounding between breeding values and THI, e.g., somehow more of the low merit sires for a trait are measured in warm/humid areas or vice versa. This association can also generate spurious G×E interaction signals when regressing breeding value on THI unless it is correctly controlled, i.e., modeling the G-E correlations as well as the G×E interaction in MRNM (Ni et al., 2019; Zhou et al., 2020). In order to estimate the spurious genetic effects of THI, we tested and estimated the spurious “heritability” of THI after adjusting it for the first 10 PCs, CG, farm locations, sex and ages (Supplementary Table 3). These adjusted THI values were used as the second trait in the MRNM to be able to fit the G-E and R-E correlations while the raw (standardized) THI values were used to model the effect of THI on the phenotypes of the main trait.

#### Estimation of Heritabilities and Genetic Correlations Between Four Carcass Traits

The genetic parameters of the four carcass traits consisting of CW, BFT, EMA and MS were estimated from a four-traits linear mixed model using genome-based restricted maximum likelihood (GREML). The four-traits linear mixed model can be written as

$$\begin{array}{c} {\text{y}}_{1}={\text{X}}_{1}{\text{b}}_{1}+{\text{Z}}_{1}{\text{g}}_{1}+{\text{e}}_{1}\\ {\text{y}}_{2}={\text{X}}_{2}{\text{b}}_{2}+{\text{Z}}_{2}{\text{g}}_{2}+{\text{e}}_{2}\\ \vdots \\ {\text{y}}_{t}={\text{X}}_{t}{\text{b}}_{t}+{\text{Z}}_{t}{\text{g}}_{t}+{\text{e}}_{t} \end{array}$$

where **y**_*i*_ is the N_*records*_ vector of observed phenotypes, **b**_*i*_ is the vector of fixed effects, **g**_*i*_ is the N_*individuals*_ vector of additive genetic, and **e**_*i*_ is N_*records*_ vector of the residual effects for the *i*th trait (*i* = 1, …, *t*). The fixed effects include 29 CG classified according to birth year and season information, 15 farm locations, 2 sex classes, ages, the first 10 PCs and a linear or/and quadratic function of THI. With other fixed effects, the linear and quadratic function of THI values and the first 10 PCs were used as fixed effects for CW and EMA, respectively (Supplementary Table 3). BFT was adjusted for quadratic function of THI and the first 10 PCs, and MS was adjusted for the first 10 PCs only (Supplementary Table 3). **X**_*i*_ and **Z**_*i*_ indicates, respectively, incidence matrix for fixed and additive genetic effects, for the *i*th trait. The variance-covariance matrix of all observed phenotypes can be written as

$$\text{var}\left(y\right)=\left[\begin{array}{ccc} {\text{Z}}_{1}\text{A}\sigma_{g_{1}}^{2}{\text{Z}}_{1}^{{}^{\prime }}+\text{I}\sigma_{e_{1}}^{2} & \cdots & {\text{Z}}_{1}\text{A}\sigma_{g_{1,t}}{\text{Z}}_{\text{t}}^{{}^{\prime }}+\text{I}\sigma_{e_{1,t}}\\ \vdots & \ddots & \vdots \\ {\text{Z}}_{t}\text{A}\sigma_{g_{1,t}}{\text{Z}}_{1}^{{}^{\prime }}+\text{I}\sigma_{e_{1,t}} & \cdots & {\text{Z}}_{\text{t}}\text{A}\sigma_{g_{t}}^{2}{\text{Z}}_{\text{t}}^{{}^{\prime }}+\text{I}\sigma_{e_{t}}^{2} \end{array}\right]$$

where **A** is the N_*individuals*_ × N_*individuals*_ genomic relationship matrix based on genome-wide SNP information (Yang et al., 2011), and **I** is an N_*records*_ × N_*records*_ identity matrix. Using GCTA software (Yang et al., 2011), the genomic relationship matrix (**A**) is computed from **A** = **WW**′/N_*SNPs*,_ where **W** is a column-standardized N_*individuals*_ × N_*SNPs*_ matrix including the genotype information of N_*individuals*_. The terms, $\sigma_{g_{i}}^{2}$ and $\sigma_{e_{i}}^{2}$, indicate the genetic and residual variance of the trait *i*, and σ_*g_ij*_ and σ_*e_ij*_ represent the genetic and residual covariances between the traits *i* and *j* (*i* = 1,*…,t*, and *j* = 1,*…,t* with *i≠j*), respectively. The random genetic and residual effects are assumed to be normally distributed with mean zero and variance $\text{A}\sigma_{g}^{2}$ and $\text{I}\sigma {}_{e}^{2}$.

#### Genotype-by-THI (G×E_THI_) Interaction Model

A univariate reaction norm model (URNM) can be used to estimate G×E_THI_ for each carcass trait of which the genetic effects are modulated by THI. The model assumes homogeneous residual variance and does not consider the correlation between carcass traits and THI values. Following Ni et al. (2019), the observed phenotype can be modeled as

$$\text{y}=\mu +\alpha_{0}+\alpha_{1}\cdot \text{c}+\text{e}$$

where μ is the mean of the adjuted phenotyeps, α_**0**_ and α_**1**_ are the N_*individuals*_ vector of the zero and first order of random regression coefficients for the random genetic effects, **c** is N_*individuals*_ vector of THI values that modulates the main phenotypes, and **e** is the residual effects. The genetic variance and covariance matrix of random regression coefficients (**K_g_**) is

(1) $${\text{K}}_{g}=\text{cov}\left(\alpha_{0},\alpha_{1}\right)=\left[\begin{array}{cc} \sigma_{\alpha_{0}}^{2} & \sigma_{\alpha_{0,1}}\\ \sigma_{\alpha_{0,1}} & \sigma_{\alpha_{1}}^{2} \end{array}\right]$$

where $\sigma_{\alpha_{0}}^{2}$ and $\sigma_{\alpha_{1}}^{2}$ are the variance of the zero and first order genetic random regression coefficients, and σ_α0,1_ is the covariance between α_**0**_ and α_**1**_, assuming that each individual has a unique covariate (i.e., THI) value such that the number of individuals is the same as the number of unique THI values. The genetic variance and covariance matrix of all individuals is a function of the matrix **K_g_** and polynomials, and can be expressed as

(2) $$\text{var}\left(g\right)=\Phi {\text{K}}_{g}\Phi^{\prime }=\left[\begin{array}{ccc} \sigma_{g_{1}}^{2} & \cdots & \sigma_{g_{1.N}}\\ \vdots & \ddots & \vdots \\ \sigma_{g_{1,N}} & \cdots & \sigma_{g_{N}}^{2} \end{array}\right]$$

where **Φ** is N_*individuals*_ × 2 matrix of the zero and first order polynomials of the THI values of N_*individuals*_, i.e., **Φ** = [**c^0^**, **c^1^**].

#### Residual-by-THI (R×E_THI_) Interaction Model

The G×E_THI_ interaction model assumes that the residual variance of main trait is homogeneous across THI values, however, this could be violated. The R×E_THI_ interaction model can capture such residual heterogeneity and can be written as

$$\text{y}=\mu +\alpha_{0}+\tau_{0}+\tau_{1}+\text{e}$$

where τ_**0**_ and τ_**1**_ are the zero and first order of random regression coefficients for residual effects. The residual effects can be modeled with random regression coefficients and the variance and covariance matrix of random regression coefficients (**K_e_**) can be represented as

(3) $${\text{K}}_{e}=\text{cov}\left(\tau_{0},\tau_{1}\right)=\left[\begin{array}{cc} \sigma_{\tau_{0}}^{2} & \sigma_{\tau_{0,1}}\\ \sigma_{\tau_{0,1}} & \sigma_{\tau_{1}}^{2} \end{array}\right]$$

where $\sigma_{\tau_{0}}^{2}$ and $\sigma_{\tau_{1}}^{2}$ are the variances of the zero and first order residual random regression coefficients, and σ_τ0,1_ is the covariance between τ_**0**_ and τ_**1**_. The residual variance and covariance matrix is a function of the matrix **K_e_** and polynomials, and can be expressed as

(4) $$\text{Var}\left(e\right)=\Phi {\text{K}}_{e}\Phi^{\prime }=\left[\begin{array}{ccc} \sigma_{e_{1}}^{2} & \cdots & \sigma_{e_{1.N}}\\ \vdots & \ddots & \vdots \\ \sigma_{e_{1,N}} & \cdots & \sigma_{e_{N}}^{2} \end{array}\right]$$

where **Φ** is N_*individuals*_ x 2 matrix of the zero and first order of the THI values of N_*individuals*_, i.e., **Φ** = [**c^0^**, **c^1^**].

#### Multivariate RNM to Estimate G×E_THI_ and R×E_THI_ Corrected for G-E_THI_ and R-E_THI_ Correlation

URNM assumes that there is no G-E_THI_ or R-E_THI_ correlation, i.e., no spurious genetic or residual correlation between the carcass traits and THI. However, it has been reported that unmodeled correlation can generate spurious G×E_THI_ or R×E_THI_ interaction signals and biased estimates (Ni et al., 2019). To avoid this bias, multivariate RNM (MRNM) can be used by jointly modeling THI as the second trait, which can account for spurious genetic and residual correlations between carcass traits and THI values (G-E_THI_ or R-E_THI_ correlations). Following Ni et al. (2019), MRNM can be written as

$$\begin{array}{c} \text{y}=\mu +\alpha_{0}+\alpha_{1}\cdot \text{c}+\tau_{0}+\tau_{1}\cdot \text{c}\\ {\text{c}}^{*}=\mu +\beta +\varepsilon \end{array}$$

where c^*^ indicates the THI values adjusted for the fixed effects (Supplementary Table 3) as the phenotypes of the second trait in the model, which can be decomposed into the grand mean, β and ε that are vectors on length N_*individuals*_ of the random spurious genetic and residual effects on THI. It is noted that spurious genetic effects for THI can be generated when there is a population stratification, which is confounded with locations that are associated with temperature and humidity. Because unmodeled spurious genetic effects of the environmental variable (and their associated correlation with the main trait) can also cause spurious G × E, we explicitly model those variance components.

The covariance between the genetic random regression coefficients of carcass traits (**α_0_** and **α_1_**) and the component of variance in THI explained by genetic effects (β) can be written as

(5) $${\text{K}}_{g,\beta }=\left[\begin{array}{c} \text{cov}\left(\alpha_{0},\beta \right)\\ \text{cov}\left(\alpha_{1},\beta \right) \end{array}\right]$$

In a similar manner, the covariance between the residual random regression coefficients of carcass traits (**τ_0_**and**τ_1_**) and residual effects of THI (ε) can be written as

(6) $${\text{K}}_{e,\varepsilon }=\left[\begin{array}{c} \text{cov}\left(\tau_{0},\varepsilon \right)\\ \text{cov}\left(\tau_{1},\varepsilon \right) \end{array}\right]$$

The variance and covariance matrix of genetic effects associated with THI values and with carcass traits can be expressed jointly in the MRNM as

$$\text{var}\left(g,\beta \right)=\left[\begin{array}{cc} \Phi {\text{K}}_{g}\Phi^{\prime } & \Phi {\text{K}}_{g,\beta }\\ {\text{K}}_{g,\beta }^{\prime }\Phi^{{}^{\prime }} & \text{var}\left(\beta \right) \end{array}\right]$$

where **K_g_** and **K**_**g**,β_ are already defined in Eqs. 1 and 5. The variance and covariance matrix of residual effects in MRNM can be expressed as

$$\text{var}\left(e,\varepsilon \right)=\left[\begin{array}{cc} \Phi {\text{K}}_{e}\Phi^{\prime } & \Phi {\text{K}}_{e,\varepsilon }\\ {\text{K}}_{e,\varepsilon }^{\prime }\Phi^{\prime } & \text{var}\left(\varepsilon \right) \end{array}\right]$$

where **K_e_** and **K**_**e**,ε_ are defined in Eqs. 3 and 6 above.

Therefore, the variance and covariance matrix of **y** and **c** can be written as

cov(y,c)=[A11σg12+σe12⋯A1Nσg1,N+σe1,N⋮⋱⋮A1Nσg1,N+σe1,N⋯ANNσgN2+σeN2A1*σg1,β+σe1,ε⋮AN*σgN,β+σeN,εA1*σg1,β+σe1,ε⋯AN*σgN,β+σeN,εAσgβ2+σeε2]

where $\sigma_{\beta }^{2}$ and $\sigma_{\varepsilon }^{2}$ are the random genetic and residual variances of THI. σ_*gi*,β_ and σ_*ei*,ε_ denote the random genetic and residual covariances between carcass trait and THI at the *i*th covariate level (*i* = 1,…,*N*). A_*i_ or **A**_i*_ indicates the *i*th column or row vector of the **A** matrix. All models described above can be fitted using MTG2.14 (Lee and van der Werf, 2016).

We compare this MRNM with the null model that is a bivariate linear mixed model fitting CW, EMA, BFT or MS as the first trait and THI as the second trait.

#### The Magnitude of Significance for G×E_THI_ and R×E_THI_ Interactions and Their Collinearity

It is often desirable to disentangle between estimated G×E_THI_ and R×E_THI_ interactions particularly when there is collinearity between these interactions that can be generated because of using the same environmental variable, i.e., THI.

The magnitude of significance for G×E_THI_ and R×E_THI_ interactions can be calculated by log-likelihood comparison between the combined G×E_THI_ and R×E_THI_ interaction model and the null model without any interaction, i.e., a function of likelihood ratio, referred to as ℳ(G×E_THI_ & R×E_THI_). In a similar manner, the magnitude of significance for the orthogonal effects of G×E_THI_ conditional on R×E_THI_, and vice versa, can be obtained by log-likelihood comparison between the combined (G×E_THI_ and R×E_THI_ interaction) model and a reduced model (with either R×E_THI_ or G×E_THI_ interaction only), referred to as ℳ(G×E_THI_|R×E_THI_) or ℳ(R×E_THI_| G×E_THI_). From these quantities, the amount of collinearity between G×E_THI_ and R×E_THI_ interactions can be approximately quantified as

$$\mathrm{The}\mathrm{magnitude}\mathrm{of}\mathrm{collinearity}=ℳ\left(\text{G}\times {\text{E}}_{\text{THI}}\&\text{R}\times {\text{E}}_{\text{THI}}\right)-ℳ\left(\text{G}\times {\text{E}}_{\text{THI}}|\text{R}\times {\text{E}}_{\text{THI}}\right)\;-ℳ\left(\text{R}\times {\text{E}}_{\text{THI}}|\text{G}\times {\text{E}}_{\text{THI}}\right)$$

## Results

### Estimated Heritabilities and Genetic Correlations Between Four Carcass Traits

In a preliminary analysis without considering interaction model, we estimated genetic variance within and covariance between four carcass traits using the four-traits linear mixed model. As shown in Supplementary Table 4, the heritability estimates for CW, BFT, EMA and MS were 0.35 (±0.02), 0.35 (±0.02), 0.33 (±0.02), and 0.42 (±0.02), respectively. A negative genetic correlation between BFT and MS was estimated (−0.03 ± 0.04) while a positive genetic correlation between EMA and MS (0.45 ± 0.03) or between CW and EMA (0.43 ± 0.03) was estimated.

### G×E_THI_ and R×E_THI_ Interactions Corrected for G-E_THI_ and R-E_THI_ Correlation

In the main analysis, we used URNM and MRNM fitting THI (see section “Materials and Methods”) to estimate G×E_THI_ and R×E_THI_ interactions for each of four carcass traits (CW, BFT, EMA and MS). We conducted various model comparisons (indexed as M1–M6 in Table 1) to obtain *p*-values for the interaction effects using likelihood ratio test, e.g., bivariate GREML as the null model vs. MRNM (M4).

**TABLE 1:** *P*-values of likelihood ratio tests for model comparisons in carcass traits analyses using THI.

Index	Model comparison	Type of interactionto be tested			CW^*a*^	BFT^*b*^	EMA^*c*^	MS^*d*^
M1	H_0_:*UnivariateGREML* H_1_:*URNMFull*	Combined G×E_THI_ and R×E_THI_	T_1_ = α_0_ + e T_1_ = α_0_ + α_1_⋅c + τ_0_ + τ_1_⋅c		2.56E–01	1.10E–01	2.53E–03	9.55E–04
M2	H_0_:*URNM*G×E H_1_:*URNMFull*	Orthogonal G×E_THI_^*e*^	T_1_ = α_0_ + τ_0_ + τ_1_⋅c T_1_ = α_0_ + α_1_⋅c + τ_0_ + τ_1_⋅c		6.78E–01	7.37E–01	1.49E–02	2.10E–01
M3	H_0_:*URNM*R×E H_1_:*URNMFull*	Orthogonal R×E_THI_^*e*^	T_1_ = α_0_ + α_1_⋅c + e T_1_ = α_0_ + α_1_⋅c + τ_0_ + τ_1_⋅c		2.22E–01	2.07E–01	4.01E–01	3.68E–04
M4	H_0_:*BivariateGREML* H_1_:*URNMFull*	Combined G×E_THI_ and R×E_THI_	T_1_ = α_0_ + e T_1_ = α_0_ + α_1_⋅c + τ_0_ + τ_1_⋅c	,T_2_ = β + ε ,T_2_ = β + ε	2.63E–02	2.31E–01	3.32E–03	3.30E–03
M5	H_0_:*MRNM*R×E H_1_:*URNMFull*	Orthogonal G×E_THI_	T_1_ = α_0_ + τ_0_ + τ_1_⋅c T_1_ = α_0_ + α_1_⋅c + τ_0_ + τ_1_⋅c	,T_2_ = β + ε ,T_2_ = β + ε	1.17E–01	7.95E–01	2.72E–02	2.46E–01
M6	H_0_:*MRNM*G×E H_1_:*URNMFull*	Orthogonal R×E_THI_	T_1_ = α_0_ + α_1_⋅c + e T_1_ = α_0_ + α_1_⋅c + τ_0_ + τ_1_⋅c	,T_2_ = β + ε ,T_2_ = β + ε	2.99E–02	3.55E–01	1.70E–01	1.60E–03

*T1 is the adjusted phenotype of main trait. T2 is adjusted THI values.*

*^*a*^CW: Carcass weight used in CW-THI interaction analysis.*

*^*b*^BFT: Back fat thickness used in BFT-THI interaction analysis.*

*^*c*^EMA: Eye muscle area used in EMA-THI interaction analysis.*

*^*d*^MS: Marbling score used in MS-THI interaction analysis.*

*^*e*^The intersection between G×E and R×E interactions indicates a dependency due to the collinearity between the two interaction effects. Orthogonal G×E or R×E interaction not includes the collinearity and can be detected from model comparison between interaction model and combined interactions model.*

**FIGURE 1:**
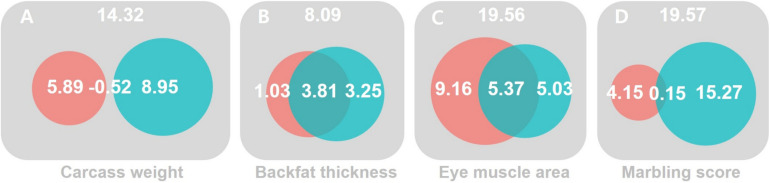
The magnitude of significance for G×E_THI_ and R×E_THI_ interactions and their collinearity. MRNM was used. The magnitude of significance for the combined G×E_THI_ (red) and R×E_THI_ (blue) interaction effects was calculated by log-likelihood comparison between the full and null models for four carcass trait; carcass weight **(A)**, back fat thickness **(B)**, eye muscle area **(C)** and marbling score **(D)** (see Supplementary Table 6). The intersection between the G×E_THI_ (red) and R×E_THI_ (blue) interaction effects indicates a dependency due to the collinearity between the two interaction effects. The magnitude of dependency (the intersection) between G×E_THI_ and R×E_THI_ interactions is the difference between the magnitude of combined G×E and R×E interactions and the sum of the magnitudes of orthogonal G×E and R×E interactions (see Methods and Supplementary Table 6).

For CW, we found no significant G×E_THI_ signal from URNM and MRNM, after adjusting for R×E_THI_ interaction (*p* = 6.78E–01 and 1.17E–01 in M2 and M5). The orthogonal R×E_THI_ interaction was not significant for URNM, after adjusting for G×E_THI_ interaction (*p* = 2.22E–01 in M3). Using MRNM, which could model the G-E correlation and G×E interaction jointly, we found a significant orthogonal R×E_THI_ interaction (*p* = 2.99E–02 in M6 in Table 1). It was noted that the significance came mainly from the component, cov(τ_1_,ε) (−0.034 ± 0.012) as shown in Supplementary Table 6. It is known that both G×E_THI_ and R×E_THI_ interactions are important in a genetic evaluation (Bohlouli et al., 2019). For these overall interaction effects, MRNM provided a significance (*p* = 2.63E–02 in M4) whereas URNM had no significance (*p* = 2.56E–01 in M1). Figure 1A shows that the G×E_THI_ and R×E_THI_ interaction effects were mostly independent (also see Supplementary Table 7).

For BFT, there was no evidence for orthogonal G×E_THI_ or R×E_THI_ interaction. Even for the combined effects of G×E_THI_ and R×E_THI_ interactions, the interaction signal was not significant in both URNM and MRNM (*p* = 1.10E–01 and 2.31E–01 in M1 and M4). The estimated G×E_THI_ or R×E_THI_ interactions were dependent to each other and there was a substantial collinearity between two estimated interactions (Figure 1B and Supplementary Tables 6, 7).

The analysis for EMA shows that the phenotypes of EMA can be modulated by THI values. In the analysis using URNM, a significant signal of G×E_THI_ interaction was discovered (*p* = 1.49E–02 in M2). We applied MRNM and also found a significance for G×E_THI_ interaction (*p* = 2.72E–02 in M5). However, R×E_THI_ interaction for EMA was not significant regardless of using URNM or MRNM (M3 and M6). The significant signal of the combined G×E_THI_ and R×E_THI_ interactions was found in MRNM (*p* = 3.32E–03 in M4). This was expected due to the large magnitude of orthogonal G×E_THI_ interaction and there was dependency between G×E_THI_ and R×E_THI_ interactions (Figure 1C and Supplementary Table 7).

Lastly, we analyzed MS and found no significant G×E_THI_ signal from URNM analysis (M2), which was consistent with the result of using MRNM (M5). The parameters of G×E_THI_ interaction components estimated from MRNM were not different from zero (Supplementary Table 6). On the other hand, we identified that the residual effects of MS can be modulated by THI, indicating a highly significant R×E_THI_ interaction from URNM and MRNM (*p* = 3.68E–04 and 1.60E–03 in M3 and M6). As shown in Supplementary Table 6, the estimated variance of R×E_THI_ interaction was significantly different from zero (0.046 ± 0.013). We also note that there was negligible residual correlation between MS and THI (r_e_ = 1.59E–03 ± 1.46E–02 in Supplementary Table 5), which agreed with non-significant estimates of cov(τ_0_,ε) and cov(τ_1_,ε) in MRNM (Supplementary Table 6). We found a significant signal of combined G×E_THI_ and R×E_THI_ interactions from MRNM (*p* = 3.30E–03 in M4), mostly due to the large magnitude of R×E_THI_ interaction (Figure 1D and Supplementary Table 7).

### When THI Are Measured Based on the Whole Growth Period

We used the last month before the slaughter day because it is reported to be the most important period to determine the phenotype of carcass traits (Kadim et al., 2004; Węglarz, 2010; Pestana et al., 2012). Nonetheless, we have tested interaction signals, considering the whole period, i.e., using the average of THI values with average maximum temperature and average relative humidity per month were obtained during the whole period (from birth to slaughter day). We found that when considering the THI values of the whole period, G×E_THI_ interactions for CW and BFT became significant, which was not significant when using the THI values of the last month only. R×E_THI_ interaction for BFT also became significant from the THI values of the whole period. However, the significant G×E_THI_ interaction for EMA with the THI values of the last month became non-significant when using the THI values of the whole period (Supplementary Table 8). This indicates that G×E_THI_ and R×E_THI_ interaction effects may be dynamically distributed across the growth trajectory, depending on traits, e.g., CW and BFT can be affected by the overall period whereas EMA can be more affected by the period near slaughter day. However, the evaluation of various THIs is beyond the scope of this study and a further study is warranted to describe this dynamic distribution of G×E across the growth period for each trait.

### Estimated Heritability From Bivariate GREML and Multivariate RNM

**FIGURE 2:**
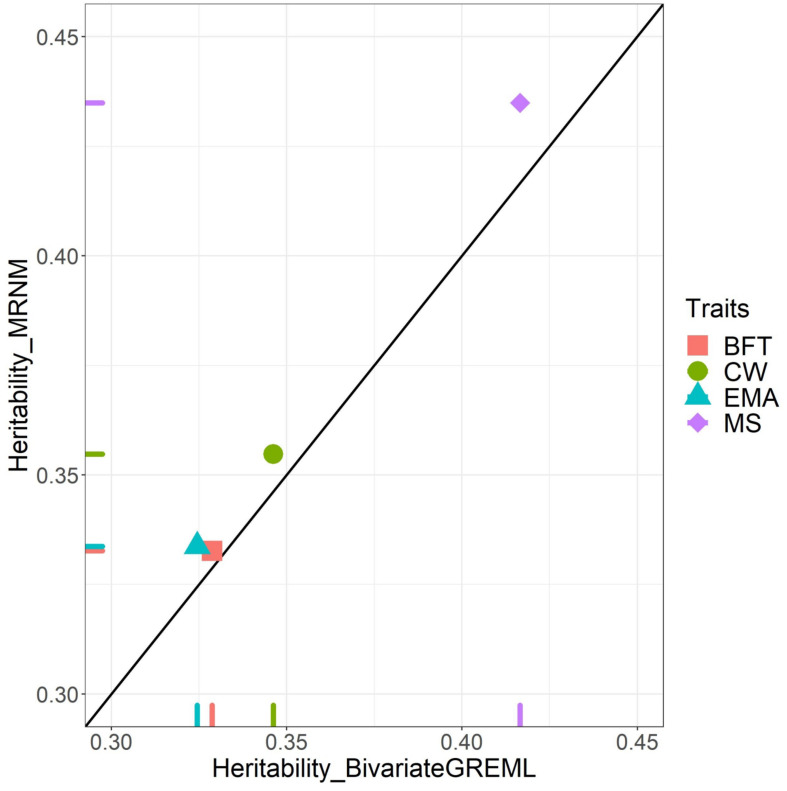
Bivariate GREML versus MRNM heritability estimates. This figure indicates the heritability difference between bivariate GREML (MRNM without GxE interaction) and MRNM with combined G×E_THI_ and R×E_THI_ for four carcass traits; carcass weight (CW), back fat thickness (BFT), eye muscle area (EMA), and marbling score (MS). *X*-axis and *Y*-axis are the heritability estimated from bivariate GREML and combined G×E_THI_ and R×E_THI_ interaction model of MRNM, respectively. MRNM with no interaction (i.e., Null model) is used as bivariate GREML. If bivariate GREML and MRNM estimates are identical, they are placed on the diagonal line. For CW, EMA, and MS, we identified that the estimated residual variances from bivariate GREML and MRNM are significantly different (*p* = 3.20E–02, 2.92E–02, and 3.37E–05 for CW, EMA, and MS based on theory (Ni et al., 2019; see Supplementary Note 1). It is noted that the estimated genetic variances are constant (Supplementary Figure 3), confirming that estimated genetic variance is invariant whether using an additive model or a non-additive interaction model (Ni et al., 2019). Therefore, the heritability differences for CW, EMA and MS were mostly due to the difference of residual variance between two models.

We estimated consistent genetic variances from bivariate GREML (the null model) and MRNM (combined G×E_THI_ and R×E_THI_ model) (Supplementary Figure 2), confirming that estimated genetic variance is invariant whether using the additive or the interaction model (Ni et al., 2019). Supplementary Figure 2 shows that for CW, EMA, and MS that had orthogonal G×E_THI_ and/or R×E_THI_ interaction, the residual variance estimated from bivariate GREML was inflated compared to MRNM, resulting in underestimated heritability (Figure 2). For EMA, estimated residual variances from bivariate GREML were significantly higher than those from MRNM (*p* = 2.92E–02 in Supplementary Table 9), which was probably due to the significant G×E_THI_ interaction effects. For CW and MS that had significant effect of R×E_THI_ interaction, the estimated residual variance from bivariate GREML also appeared to be inflated, compared to MRNM (*p* = 3.20E–02 and 3.37E–05). The residual variances from bivariate GREML and MRNM for BFT were not significantly different from one another (*p* = 3.73E–01 in Supplementary Table 9). It was also observed that the ranks of estimated breeding values were changed between the best and null model for EMA, CW and MS that showed significant interactions (Supplementary Figure 3). Based on the simulation data of the previous study (Ni et al., 2019), the estimated genetic variance is invariant whether the interactions exist or not, therefore the heritability difference is mostly due to the difference of residual variances between two models, which was also observed in this study (Figure 2 and Supplementary Figure 2).

## Discussion

In this study, we used MRNM to estimate genetic and non-genetic variance components that were changed with respect to THI values, accounting for G-E_THI_ and R-E_THI_ correlations correctly. From the MRNM analyses, we show that there are significant effects of R×E_THI_ interactions for CW that are orthogonal to each other. For BFT, there is not any interactions and they are not orthogonal to one another. We also show for EMA and MS that there are significant combined G×E_THI_ and R×E_THI_ interactions, where EMA has a significant signal for orthogonal G×E_THI_ whereas MS has a significance for orthogonal R×E_THI_. We found that interactions are ubiquitous among carcass traits, indicating that it is necessary to include interaction components in the genetic evaluation of Hanwoo cattle.

Our results indicate that inappropriate models would detect inflated interaction signals and produce biased estimates, which agrees with previous studies (Schnyder et al., 2001; Ni et al., 2019). For example, there was a notable difference between URNM and MRNM in the detection of interactions for CW, that is, the combined G×E_THI_ and R×E_THI_ interaction and the orthogonal R×E_THI_ interaction were detected from MRNM only. It is also noted that the significance of URNM tends to be different from MRNM, given the results of *p* = 2.22E–01 (URNM) and 2.99E–02 (MRNM) for orthogonal R×E_THI_ interaction on CW, and *p* = 1.49E–02 (URNM) and 2.72E–02 (MRNM) for G×E_THI_ interaction on EMA. This difference was probably due to the fact that MRNM is better to disentangle interaction effects from (G-E and R-E) correlations between the traits (CW and EMA) and THI (Ni et al., 2019). Furthermore, without considering R×E_THI_ interaction appropriately, G×E_THI_ can be inflated as two kinds of interactions can be confounded in a G×E analysis without modeling R×E_THI_ (Supplementary Table 1; Ni et al., 2019). The R×E_THI_ interaction may be generated by changing farm environment factors, such as a decrease in feed intake and feed efficiency, related to THI values (Young, 1983; Piao and Baik, 2015; Sejian et al., 2018). In our analyses using MRNM, we have modeled G×E_THI_ and R×E_THI_ jointly and showed that G×E_THI_ and R×E_THI_ interactions were successfully estimated on CW, EMA, and MS.

THI values can be used as discrete variable by dividing the samples arbitrarily into multiple groups, e.g., the quartile of THI levels (Brügemann et al., 2011; Bohlouli et al., 2019). Although Jaffrezic et al. (2000) reported that estimates from models fitting continuous and discrete THI values were not statistically different, it is obvious that individual differences in THI values within each discrete group are ignored, which can result in decreasing the power to detect the G×E interaction. In our analysis, we used continuous THI values, which is likely to produce more reliable estimates, compared to when using arbitrary discrete THI values.

The estimated genetic correlation between the traits and THI may be due to confounding between the genetic effects and environmental factors such as farm location (or some unknown factors), probably not due to the genuine genetic effects of THI. Although this genetic correlation is not biological correlation, this also can cause spurious G×E signals as like G-E correlation would cause. Unless this correlation is correctly accounted for, it is possible to get biased interaction signals. This problem is likely to be reduced when applying MRNM that can disentangle interactions from any confounding correlation.

In the presence of interactions, the estimated heritability in bivariate GREML was biased due to unmodeled G×E and R×E interactions. This result agrees with previous studies, e.g., Bohlouli et al. (2019) reported that neglecting G×E interaction results in an underestimated heritability in a simulation study. Moreover, Ni et al. (2019) and Zhou et al. (2020) showed that biased heritability can be estimated when R×E interaction is ignored as well.

There are a number of limitations in this study. First, we used a single environmental variable only, THI, although there can be multiple (unknown) environmental variables that can increase the proportion of phenotypic variance explained by interaction effects. Second, the covariance structure used to estimate R×E interaction in this study was based on an identity matrix because there were no repeated measures for the animals. It is desirable to collect repeated measures or construct the covariance structure for R×E interaction based on the product of multiple environmental variables, e.g., an environmental relationship matrix, in a further study, which can increase the power to estimate R×E interaction. Third, only the first order of G×E or R×E interaction was considered in this study, and a further study is required to validate our findings with higher order interactions. Fourth, because the period around the slaughter date is most important for the quality of carcass traits (Kadim et al., 2004; Węglarz, 2010; Pestana et al., 2012; Hagenmaier et al., 2016), we did not explicitly evaluate the significance of interactions with THI values measured on each month from the slaughter although we show the results with the averaged THI of the whole period. Fifth, we did not access specific information about the distance from the slaughterhouse, types of transport, transport time and road condition. Lastly, the model used in this study does not estimate causality and a prior information about the causality is essentially required.

## Conclusion

In conclusion, the phenotypic variance of the carcass traits of Hanwoo can be modulated by THI, revealing a novel genetic and environmental architecture of the traits. For estimating G×E interaction, MRNM is a flexible model that can accommodate a continuous environmental variable such as THI, and correctly account for confounding effects from R×E interaction as well as G-E and R-E correlations. We report that there are significant G×E_THI_ interaction for EMA and significant R×E_THI_ interactions for CW and MS, which has an important implication in the genetic evaluation of the traits (Jaffrezic et al., 2000; Schnyder et al., 2001). Because of these significant interactions, the estimated heritability of additive models can be biased, suggesting that THI information should be used. Our results are based on THI measured in the last month of the growth period, which can be extend to each month in the whole period. We argue that the estimation of genetic parameters should be based on appropriate models to avoid any potential bias of estimates. These results highlight finding of the novel genetic and environmental architecture in beef cattle and should serve as a basis for future studies aiming at estimation and genomic prediction.

## Data Availability Statement

The data analyzed in this study was obtained from the National Institute of Animal Science in South Korea, the following licenses/restrictions apply: the data is proprietary and cannot be released publicly. Requests to access these datasets should be directed to DL, lim.dj@korea.kr.

## Ethics Statement

The current study was approved by the Animal Care and Use Committee of the National Institute of Animal Science (NIAS), Rural Development Administration (RDA), South Korea.

## Author Contributions

YC, SHoL, and SHwL conceived the idea. SHoL and SHwL directed and supervised the study. H-KL and DL organized the database. YC and SHoL wrote the first draft of the manuscript. YC, SHoL, SHwL, and JW contributed to manuscript revision. All authors contributed to read and approved the submitted version.

## Conflict of Interest

The authors declare that the research was conducted in the absence of any commercial or financial relationships that could be construed as a potential conflict of interest.
