# Supplementary material for: THI Modulation of Genetic and Non-genetic Variance Components for Carcass Traits in Hanwoo Cattle
Source: Front Genet. 2020 Dec 23;11:576377. doi: 10.3389/fgene.2020.576377 (PMC7786192; doi:10.3389/fgene.2020.576377)
Supplement: Supplementary file 1 [file Data_Sheet_1.docx]

**Supplementary Table 1. A review of G**$\boldsymbol{\times}$**E interaction analysis in livestock**

| **Authors** | **Breeds** | **Published years** | **Traits** | **Environmental variables^a^** | **Models^b^** | **Modelling residual heterogeneity (R**$\boldsymbol{\times}$**E interaction)^c^** | **Modelling G-E correlation^d^** |
| --- | --- | --- | --- | --- | --- | --- | --- |
| T. Ibi et al | Japanese Black cattle | 2005 | Carcass traits | Regions (cold, warm) | Bivariate GREML | **No** | **No** |
| D.G.F. Guidolin et al | Nellore | 2012 | Body weight | Region (Tropical) | Bivariate GREML | **No** | **No** |
| M. L. Santana Jr et al | Brazilian; Nellore, Brangus, Tropical composite (crossbreed) | 2015 | WW, YW | ACTHI | URNM | **No** | **No** |
| H. L. Bradford et al | Angus | 2016 | WW, YW | THI | URNM | **No** | **No** |
| Roberto Carvalheiro et al | Nellore | 2019 | PWG | Climate condition  (harsh, average, good) | URNM | Yes | **No** |
| F. Jaffrezic et al | Holstein | 2001 | Lactation | Time | URNM | Yes | **No** |
| K. Brugemann et al | Holstein | 2011 | MY, SCC | DIM, THI | URNM  Bivariate GREML | Yes | **No** |
| Mario L. Santana Jr et al | Brazilian Holstein | 2016 | MY, SCC | DIM, THI | URNM | **No** | **No** |
| M. Bohlouli et al | German Holstein | 2019 | MY, SCC | DIM, THI | URNM | **No** | **No** |
| SeokHyun Lee et al | Korean Holstein | 2019 | MY | THI | URNM | **No** | **No** |
| Urs Schnyder et al | Pig | 2000 | Feed intake | Day in milk | URNM | Yes | **No** |

**^a^**Environmental variables used in the G$\boldsymbol{\times}$E study.

**^b^**Models used in the G$\boldsymbol{\times}$E study.

**^c^**Unmodeled residual heterogeneity ($R\boldsymbol{\times}E$ interaction) can result in biased estimates of genetic parameters including G$\boldsymbol{\times}$E interaction.

**^d^**Unmodeled G-E correlation (genetic and residual correlations (or confounding) between the main phenotypes and environmental can result in biased estimates of G$\boldsymbol{\times}$E interaction. In the previous literatures, G$\boldsymbol{\times}$E interaction has been detected using bivariate GREML or URNM. However, while genetic correlation between the trait and environmental variables existed and the model without considering this correlation can cause the unbiased residual variance, there were few or no studies to consider correlation and interaction simultaneously. Hence, modelling correlation and interaction jointly is necessary to detect and estimate G$\boldsymbol{\times}$E interaction more accurately.

**Supplementary Table 2. Summary statistics for carcass traits**

| **Traits** | **Number of animals** | **Mean** | **SD** | **Min** | **Max** |
| --- | --- | --- | --- | --- | --- |
| **CW (kg)** | 9243 | 442.1 | 50.1 | 216 | 624 |
| **BFT (mm)** | 9202 | 14.1 | 4.6 | 2 | 30 |
| **EMA (cm^2^)** | 9241 | 95.8 | 11.5 | 51 | 136 |
| **MS (1-9)** | 9317 | 6.2 | 1.8 | 1 | 9 |

Phenotypic data outside three standard deviations of the population mean was excluded, and the number of animals used was 9243, 9202, 9241 and 9317 for carcass weight (CW), back fat thickness (BFT), eye muscle area (EMA), and marbling scores (MS). The summary statistics for carcass traits indicate mean, standard deviation (SD), minimum (Min), and maximum (Max) of the used data.

| **Factors** | **CW** | **BFT** | **EMA** | **MS** | **THI** |
| --- | --- | --- | --- | --- | --- |
| Model1. Y = CG $+$ Sex $+$ Farm location $+$ Age $+$ e | 98698.37 | 56235.77 | 72220.68 | 36840.53 | 55792.59 |
| Model2. Y = CG $+$ Sex $+$ Farm location $+$ Age $+$ THI $+$ e | 98686.01 | 56220.14 | 72220.85 | 36842.44 |  |
| Model3. Y = CG $+$ Sex $+$ Farm location $+$ Age $+$ THI*THI $+$ e | 98687.64 | 56219 | 72221.32 | 36842.51 |  |
| Model4. Y = CG $+$ Sex $+$ Farm location $+$ Age $+$ THI + THI*THI + e | 98685.21 | 56220.21 | 72220.58 | 36842.6 |  |
| Model5. Y = CG $+$ Sex $+$ Farm location $+$ Age $+$ first 10 PCs $+$ e | 97903.02 | 55834.12 | 71846.33 | 36693.54 | 55760.88 |
| Model6. Y = CG $+$ Sex $+$ Farm location $+$ Age $+$ THI $+$ first 10 PCs $+$ e | 97888.89 | 55823.89 | 71844.53 | 36694.91 |  |
| Model7. Y = CG $+$ Sex $+$ Farm location $+$ Age $+$ THI*THI $+$ first 10 PCs $+$ e | 97891.07 | 55822.73 | 71845.44 | 36695.19 |  |
| Model8. Y = CG $+$ Sex $+$ Farm location $+$ Age $+$ THI + THI*THI + first 10 PCs $+$ e | 97886.19 | 55823.34 | 71842.39 | 36694.03 |  |

**Supplementary Table 3. AIC value comparison to select fixed effects for carcass traits**

Four linear models were compared to select fixed effects using Akaike’s information criteria (AIC). Y indicates each trait, and sex, CG (contemporary group; birth year, birth season), farm location and age were included in all models. The first 10 principal components (PCs) are included in Model5 - 8. The phenotypes of CW and EMA were adjusted for linear and quadratic effects of THI as well as first 10 PCs, while BFT was adjusted for quadratic effects of THI and first 10 PCs. MS was adjusted for first 10 PCs without any THI effects because the null model without THI had the lowest AIC values.

**Supplementary Table 4. Heritability (diagonal), phenotypic correlation (above the diagonal) and genetic correlation (below the diagonal) between carcass traits**

|  | **CW** | **BFT** | **EMA** | **MS** |
| --- | --- | --- | --- | --- |
| **CW** | 3.48.E-01 $\pm$ 1.72.E-02 | 3.72.E-01 $\pm$ 9.62.E-03 | 4.88.E-01 $\pm$ 9.04.E-03 | 1.81.E-01 $\pm$ 1.02.E-02 |
| **BFT** | 2.02.E-01 $\pm$ 4.11.E-02 | 3.35.E-01 $\pm$ 1.78.E-02 | 8.92.E-02 $\pm$ 1.03.E-02 | 1.23.E-01 $\pm$ 1.03.E-02 |
| **EMA** | 4.28.E-01 $\pm$ 3.49.E-02 | -1.51.E-01 $\pm$ 4.56.E-02 | 3.31.E-01 $\pm$ 1.73.E-02 | 3.66.E-03 $\pm$ 1.04.E-02 |
| **MS** | 1.24.E-01 $\pm$ 3.98.E-02 | -2.55.E-02 $\pm$ 4.21.E-02 | 4.50.E-01 $\pm$ 3.36.E-02 | 4.16.E-01 $\pm$ 1.73.E-02 |

Heritability and genetic (phenotypic) correlation were estimated between four carcass traits; carcass weight (CW), backfat thickness (BFT), eye muscle area (EMA) and marbling score (MS). The estimated heritability for THI is 5.43E-02 (1.06E-02). This four-traits model was used for a preliminary analysis only, which is not involved in the model comparisons in the main interaction analysis.

**Supplementary Table 5. Genetic and residual correlations between carcass traits and THI variables**

| **THI** | **CW** | **BFT** | **EMA** | **MS** |
| --- | --- | --- | --- | --- |
| $r_{g}$ | -8.50E-02 $\pm$ 8.74E-02 | -1.45E-01 $\pm$ 9.09E-02 | 1.54E-01 $\pm$ 8.71E-02 | -4.21E-02 $\pm$ 8.37E-02 |
| $r_{e}$ | 1.28E-02 $\pm$ 1.41E-02 | 2.50E-02 $\pm$ 1.41E-02 | -2.04E-02 $\pm$ 1.40E-02 | 1.59E-03 ± 1.46E-02 |

Genetic correlation ($r_{g}$) and residual correlation ($r_{e}$) between carcass traits and THI variables were estimated; carcass weight (CW), backfat thickness (BFT), eye muscle area (EMA) and marbling score (MS). The estimated heritability for THI is 5.43E-02 (1.06E-02).

**Supplementary Table 6. (Co)variance estimated from MRNM for carcass traits**

| **Parameter** | **CW** | **BFT** | **EMA** | **MS** |
| --- | --- | --- | --- | --- |
| $Var(\alpha_{0})$ | 0.3542 $\pm$ 0.0217 | 0.3397 $\pm$ 0.0223 | 0.3314 $\pm$ 0.0213 | 0.4272 $\pm$ 0.0232 |
| $Var(\alpha_{1})$ | 0.0038 $\pm$ 0.0042 | 0.0023 $\pm$ 0.0038 | 0.0077 $\pm$ 0.0052 | 0.0021 $\pm$ 0.0039 |
| $Cov(\alpha_{0}, \alpha_{1})$ | -0.0021 $\pm$ 0.0093 | 0.0049 $\pm$ 0.0093 | 0.0245 $\pm$ 0.0095 | 0.0177 $\pm$ 0.0097 |
| $Var(\beta)$ | 0.0496 $\pm$ 0.0106 | 0.0502 $\pm$ 0.0107 | 0.0516 $\pm$ 0.0108 | 0.0509 $\pm$ 0.0108 |
| $Cov(\alpha_{0}, \beta)$ | -0.0103 $\pm$ 0.0116 | -0.0194 $\pm$ 0.0118 | 0.0201 $\pm$ 0.0115 | -0.0049 $\pm$ 0.0123 |
| $Cov(\alpha_{1}, \beta)$ | 0.0126 $\pm$ 0.0053 | 0.0032 $\pm$ 0.0052 | 0.0059 $\pm$ 0.0057 | 0.0054 $\pm$ 0.0052 |
| $Var(\tau_{0})$ | 0.6443 $\pm$ 0.0195 | 0.6814 $\pm$ 0.0204 | 0.6617 $\pm$ 0.0197 | 0.5551 $\pm$ 0.0183 |
| $Var(\tau_{1}$) | 0.0233 $\pm$ 0.0136 | 0.009 $\pm$ 0.0138 | 0.0192 $\pm$ 0.0138 | 0.0455 $\pm$ 0.0132 |
| $Cov(\tau_{0}, \tau_{1})$ | 0.0001 $\pm$ 0.0084 | 0.0149 $\pm$ 0.0086 | 0.0018 $\pm$ 0.0087 | -0.0081 $\pm$ 0.0082 |
| $Var(\varepsilon$) | 0.9513 $\pm$ 0.0162 | 0.9508 $\pm$ 0.0163 | 0.9498 $\pm$ 0.0163 | 0.9503 $\pm$ 0.0163 |
| $Cov(\tau_{0}, \varepsilon)$ | 0.0105 $\pm$ 0.0112 | 0.0204 $\pm$ 0.0115 | -0.0161 $\pm$ 0.0113 | 0.002 $\pm$ 0.011 |
| $Cov(\tau_{1}, \varepsilon)$ | -0.0344 $\pm$ 0.0119 | -0.0058 $\pm$ 0.0118 | -0.0253 $\pm$ 0.012 | 0.0004 $\pm$ 0.0117 |

The variance and covariance were estimated from MRNM (combined $G\times E_{\mathrm{THI}}$ and $R\times E_{\mathrm{THI}}$ model) for carcass traits; carcass weight (CW), back fat thickness (BFT), eye muscle area (EMA) and marbling score (MS). The combined $G\times E_{\mathrm{THI}}$ and $R\times E_{\mathrm{THI}}$ interaction model of MRNM (MRNM combined model) was used in order to estimate the unbiased variances in the traits that have interaction effect.

**Supplementary Table 7. The likelihood ratios for combined and orthogonal** $\boldsymbol{G\times}\mathbf{E}_{\mathbf{THI}}$ **and** $\boldsymbol{R\times}\mathbf{E}_{\mathbf{THI}}$ **interactions and their collinearity using MRNM**

| **Index used in Table 1** | **Model comparison** | **Type of interaction** | **CW** | **BFT** | **EMA** | **MS** |
| --- | --- | --- | --- | --- | --- | --- |
| M4 | $H_{0} :Bivariate GREML$  $H_{1} :MRNM Full$ | Combined  $G\times E_{\mathrm{THI}}$ and $R\times E_{\mathrm{THI}}$ | 14.32 | 8.09 | 19.56 | 19.57 |
| M5 | $H_{0} :MRNM R\boldsymbol{\times}E$_THI_  $H_{1} :MRNM Full$ | Orthogonal $G\times E_{\mathrm{THI}}$ | 5.89 | 1.03 | 9.16 | 4.15 |
| M6 | $H_{0} :MRNM G\boldsymbol{\times}E$_THI_  $H_{1} :MRNM Full$ | Orthogonal R$\times E_{\mathrm{THI}}$ | 8.95 | 3.25 | 5.03 | 15.27 |
|  |  | Dependency or collinearity | -0.52 | 3.81 | 5.37 | 0.15 |

The magnitude of significance for $G\boldsymbol{\times}E_{\mathrm{THI}}$ and $R\boldsymbol{\times}E_{\mathrm{THI}}$ interactions was calculated by log-likelihood comparison between the combined $G\times E_{\mathrm{THI}}$ and $R\times E_{\mathrm{THI}}$ interaction model (MRNM Full) and the null model without any interaction (bivariate GREML), i.e. a function of likelihood ratio, referred to as $\mathcal{M(}G\times E_{\mathrm{THI}} \& R\times E_{\mathrm{THI}})$. In a similar manner, the magnitude of significance for the orthogonal effects of $G\times E_{\mathrm{THI}}$ or $R\times E_{\mathrm{THI}}$ was obtained by log-likelihood comparison between the MRNM Full model and a reduce model (MRNM $R\times E_{\mathrm{THI}}$ or MRNM $G\times E_{\mathrm{THI}}$), referred to as $\mathcal{M(}G\times E_{\mathrm{THI}} | R\times E_{\mathrm{THI}})$ or $\mathcal{M(}R\times E_{\mathrm{THI}} | G\times E_{\mathrm{THI}})$. From these quantities, the amount of collinearity between $G\times E_{\mathrm{THI}}$ and $R\times E_{\mathrm{THI}}$ interactions was approximately quantified as, t$he magnitude of collinearity=\mathcal{M(}G\times E_{\mathrm{THI}} \& R\times E_{\mathrm{THI}})-\mathcal{M(}G\times E_{\mathrm{THI}} | R\times E_{\mathrm{THI}}\mathcal{)- M(}R\times E_{\mathrm{THI}} | G\times E_{\mathrm{THI}})$.

**Supplementary Table 8. P-values of likelihood ratio tests for model comparisons in carcass traits analyses using THI of the whole period*^a^***

| **Model comparison** | **Type of interaction**  **to be tested** |  | $\mathbf{CW}^{\boldsymbol{b}}$ | $\mathbf{BFT}^{\boldsymbol{c}}$ | $\mathbf{EMA}^{\boldsymbol{d}}$ | $\mathbf{MS}^{\boldsymbol{e}}$ |
| --- | --- | --- | --- | --- | --- | --- |
| $H_{0} :Bivariate GREML$  $H_{1} :MRNM Full$ | Combined  $G\times E_{\mathrm{THI}}$ and $R\times E_{\mathrm{THI}}$ | $T_{1}= \alpha_{0}+e$ , $T_{2}= \beta+\varepsilon$  $T_{1}= \alpha_{0}+\alpha_{1}\cdot c+\tau_{0}+\tau_{1}\cdot c$ ,$T_{2}= \beta+ \varepsilon$ | 1.83E-04 | 8.71E-13 | 2.09E-01 | 3.48E-13 |
| $H_{0} :MRNM R\times E$  $H_{1} :MRNM Full$ | Orthogonal $G\times E_{\mathrm{THI}}$ | $T_{1}= \alpha_{0}+\tau_{0}+\tau_{1}\cdot c$ ,$T_{2}= \beta+ \varepsilon$  $T_{1}= \alpha_{0}+\alpha_{1}\cdot c+\tau_{0}+\tau_{1}\cdot c$ ,$T_{2}= \beta+ \varepsilon$ | 5.49E-03 | 1.01E-02 | 8.34E-02 | 8.66E-02 |
| $H_{0} :MRNM G\times E$  $H_{1} :MRNM Full$ | Orthogonal $R\times E_{\mathrm{THI}}$ | $T_{1}= \alpha_{0}+\alpha_{1}\cdot c+e$ ,$T_{2}= \beta+ \varepsilon$  $T_{1}= \alpha_{0}+\alpha_{1}\cdot c+\tau_{0}+\tau_{1}\cdot c$ ,$T_{2}= \beta+ \varepsilon$ | 4.18E-03 | 1.31E-05 | 1.88E-01 | 2.92E-05 |

Note: T1 is the adjusted phenotype of main trait. T2 is adjusted THI of the whole period

^a^THI of the whole period: the average of THI values with average maximum temperature and average relative humidity per month were obtained during the whole period (from birth to slaughter day)

^b^CW: Carcass weight used in CW-THI interaction analysis

^c^BFT: Back fat thickness used in BFT-THI interaction analysis

^d^EMA: Eye muscle area used in EMA-THI interaction analysis

^e^MS: Marbling score used in MS-THI interaction analysis

We detected the significant $G\boldsymbol{\times}E_{\mathrm{THI}}$ or/and $R\boldsymbol{\times}E_{\mathrm{THI}}$ interactions for four carcass trait using THI of the whole period (from birth to slaughter day). Compared to the results with THI of the last month only, $G\boldsymbol{\times}E_{\mathrm{THI}}$ for CW and BFT became significant, and $R\boldsymbol{\times}E_{\mathrm{THI}}$ for BFT also became significant. However, the significant signal of $G\boldsymbol{\times}E_{\mathrm{THI}}$ for EMA with THI of the last month became non-significant.

**Supplementary Table 9. Significant difference in residual variance between bivariate** **GREML^a^ and MRNM^b^**

| **Trait** | **Difference^c^** | **SE^d^** | **h^2^(bivariate GREML)** | **h^2^(MRNM)** | **p-value^e^** |
| --- | --- | --- | --- | --- | --- |
| CW | -0.0262 | 0.0001 | 0.3462 | 0.3547 | 3.20E-02 |
| BFT | -0.0115 | 0.0002 | 0.3287 | 0.3327 | 3.73E-01 |
| EMA | -0.0268 | 0.0002 | 0.3245 | 0.3337 | 2.92E-02 |
| MS | -0.0463 | 0.0001 | 0.4166 | 0.4349 | 3.37E-05 |

**^a^**Alternative model (H1) of M4 in Table 1

**^b^**Null model (H0) of M4 in Table 1

**^c^**Difference = the residual variance estimated from MRNM – the residual variance estimated from bivariate GREML

**^d^**Standard error of the difference was calculated based on the theory in Supplementary Note 1

**^e^**p-value was obtained based a Wald test using the difference of residual variances and its SE

We confirmed if the residual variance between multivariate models was statistically different based on Supplementary Note 1. The multivariate models are bivariate GREML, which is bivariate linear mixed model, and MRNM with both $G\boldsymbol{\times}E_{\mathrm{THI}}$ and $R\boldsymbol{\times}E_{\mathrm{THI}}$ interactions where the first traits is one of carcass traits and the second trait is the adjusted THI values. The significant differences were identified for some carcass traits that have $G\boldsymbol{\times}E_{\mathrm{THI}}$ and/or $R\boldsymbol{\times}E_{\mathrm{THI}}$ interaction; carcass weight (CW), backfat thickness (BFT), eye muscle area (EMA) and marbling score (MS). According to the constant genetic variance regardless of modelling G$\boldsymbol{\times}$E interaction in statistical model (Supplementary Figure 2) and above results, the heritability estimates for all traits from MRNM were significantly higher than those of bivariate GREML (Figure 2).


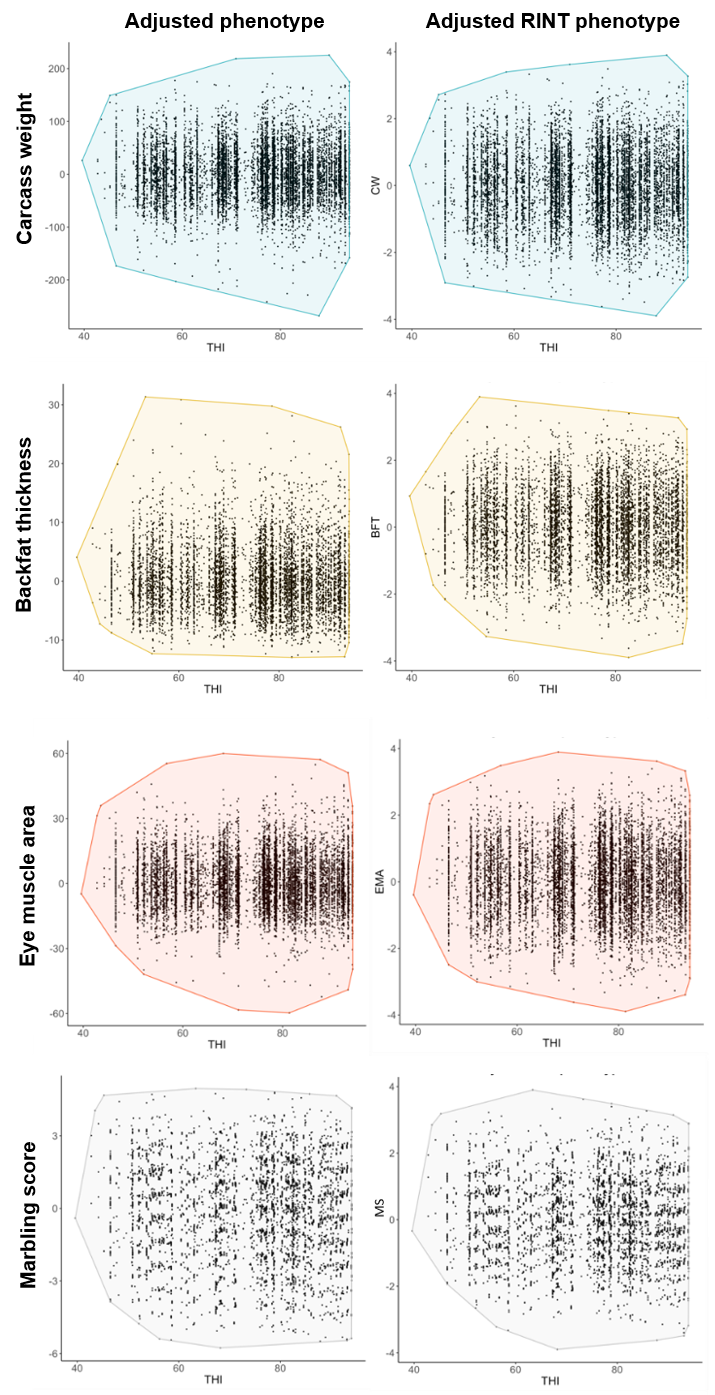


**Supplementary Figure 1. Distributions according to the type of observed phenotype**

A change in the distribution of observed phenotype after rank-based inverse transformation (RINT). X-axis and Y-axis indicate THI variables and the observed phenotype of carcass traits, respectively. For adjusted phenotype of backfat thickness and marbling score, the normality assumption is violated. However, after RINT, they follow the normality.


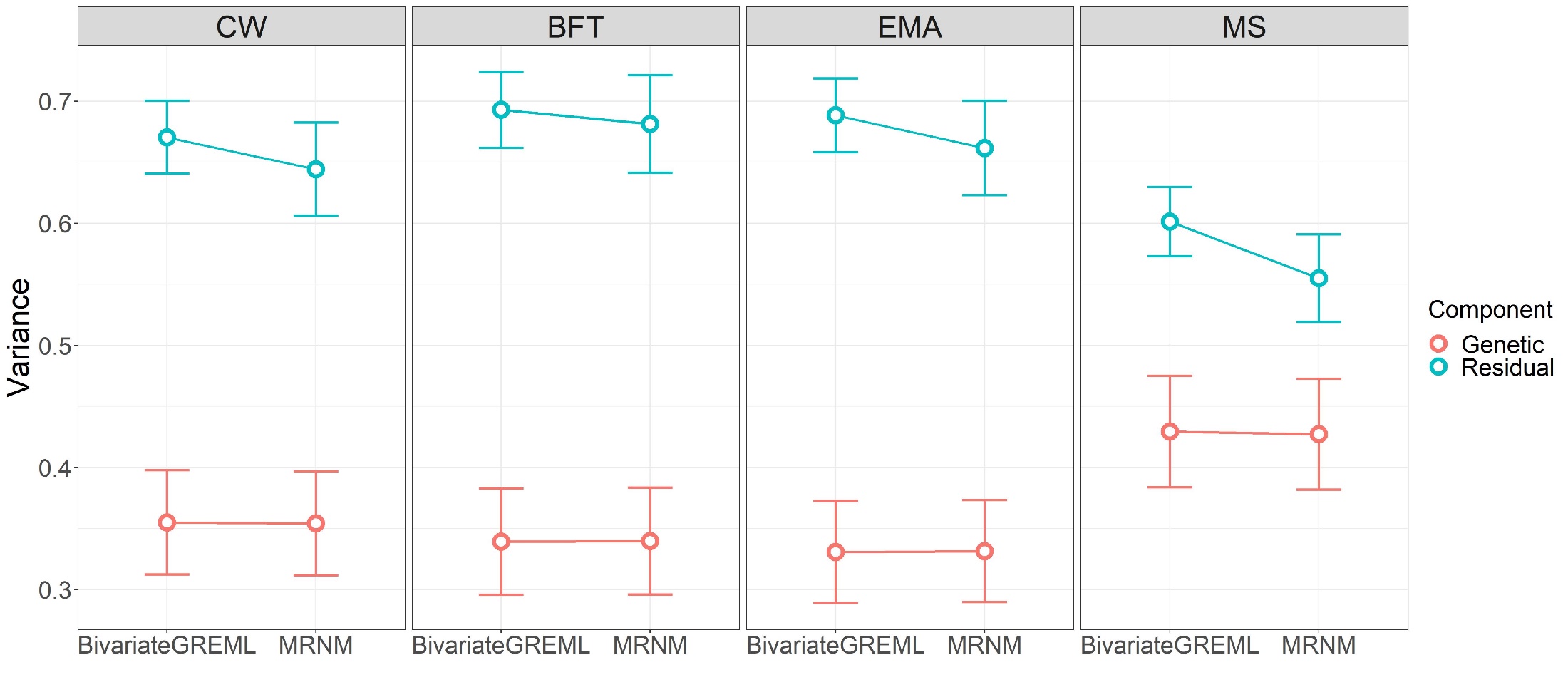


**Supplementary Figure 2. Comparison of genetic and residual variances estimated from bivariate GREML and MRNM**

Estimated genetic and residual variances of carcass weight (CW), back fat thickness (BFT), eye muscle area (EMA), and marbling score (MS) are compared between bivariate GREML (MRNM with no interaction) and MRNM (MRNM with combined $G\times E_{\mathrm{THI}}$ and $R\times E_{\mathrm{THI}}$). The error bars are the 95% confidence intervals of the estimates.


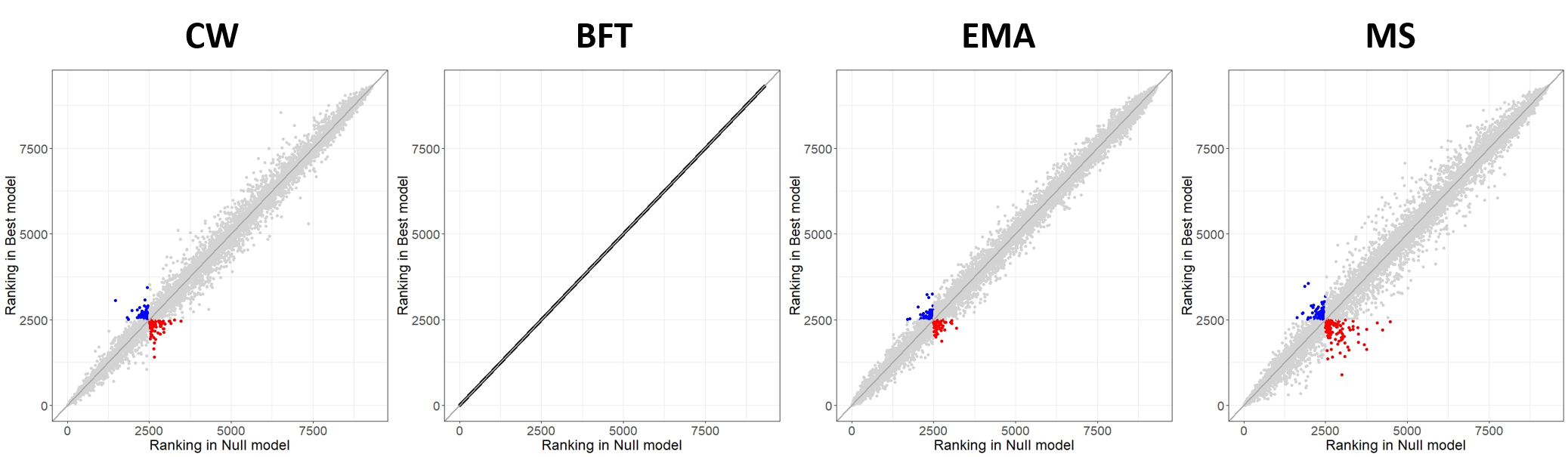


**Supplementary Figure 3. Ranking of estimated breeding values changes between bivariate GREML and MRNM**

We compared the rank of predicted phenotypes between the null (bivariate GREML) and the best model for CW, BFT, EMA and MS in the main interaction analyses. For example, when selecting the top 2500 animals, the blue coloured individuals, which should be selected according to their predicted phenotypic values from the best model, were excluded with the null model. The predicted phenotypic values for the null model was calculated using estimated main genetic effects (i.e. breeding values), i.e. $\hat{y}$ = $\hat{\alpha_{0}}$ and those for the best models were calculated using estimated main genetic and interaction effects for individuals, given THI values, e.g. $\hat{y}= \hat{\alpha_{0}}+ \hat{\alpha_{1}} \times THI$ for CW. The x axis represents the rank of predicted phenotypes from the null model (bivariate GREML) and the y axis indicates the rank of predicted phenotypes from the best model. The dark grey line is x = y, and red and blue coloured animals are switched to be selected in or out, depending on the models. The number of switched animals (coloured) was 89, 79, 113 or 0 for CW, EMA, MS or BFT that had no significant interaction.

**Supplementary Note 1. Testing a statistical difference between estimated residual variance from bivariate GREML and MRNM**

Following Ni et al (2019), we tested if the residual variances from bivariate GREML and MRNM were statistically different. Assuming that true model is MRNM which consists of $y_{i}$ = $\alpha_{i0}$ + $\alpha_{i1}\cdot c_{i}$ + $e_{i}$ and $c_{i}$ = $\beta_{i}$ + $\varepsilon_{i}$, the estimated residual variance can be unbiased, but the general model such as bivariate GREML may overestimate the residual variance that contain the interactions, i.e. $y_{i}$ = $\alpha_{i0}$ + $e_{i}^{*}$ and $c_{i}$ = $\beta_{i}$ + $\varepsilon_{i}$, where $e_{i}^{*}$ = $\alpha_{i1}\cdot c_{i}$ + $e_{i}$. Based on this assumption, the sampling variance of the difference between bivariate GREML and MRNM on residual variance can be estimated and an equation can be expressed as

$\sigma_{d}^{2}= \sigma_{X}^{2}+ \sigma_{Y}^{2}- 2\cdot cov(\mathbf{X},\mathbf{Y})$ (Eq1)

where X is the residual variance from MRNM ($\hat{\sigma}_{e}^{2}$) and Y is the residual variance from bivariate GREML ($\hat{\sigma}_{e*}^{2}$). $\sigma_{X}^{2}$ and $\sigma_{Y}^{2}$ are the sampling variance of X and Y, respectively.

From MRNM, the assumption is that the mean of estimated residual values is zero, and the estimated residual variance is expressed as $\mathbf{X}= \hat{\sigma}_{e}^{2}=E(\hat{e}^{2})$. From bivariate GREML, the estimated residual variance is expressed as $\mathbf{Y}= \hat{\sigma}_{e*}^{2}=E[\left( {\hat{\boldsymbol{\alpha}}}_{\boldsymbol{1}}\cdot\mathbf{c} + \hat{\mathbf{e}} \right)$^2^]$=E\left( {\hat{\mathbf{e}}}^{\mathbf{2}} \right)+E\left( {\hat{\boldsymbol{\alpha}}}_{\boldsymbol{1}}\cdot\mathbf{c}^{\boldsymbol{2}} \right)+ E\left( {\hat{\boldsymbol{\alpha}}}_{\boldsymbol{1}}\cdot\mathbf{c}^{\boldsymbol{2}}\boldsymbol{\cdot}{\hat{\mathbf{e}}}^{\mathbf{2}} \right)$. Hence, Y can be written as a linear function of **X** as **Y** = **X**b + $\lambda$ where b is a regression coefficient (b = 1) and $\lambda$ = $E\left( {\hat{\boldsymbol{\alpha}}}_{\boldsymbol{1}}\cdot\mathbf{c}^{\boldsymbol{2}} \right)+ E\left( {\hat{\boldsymbol{\alpha}}}_{\boldsymbol{1}}\cdot\mathbf{c}^{\boldsymbol{2}}\boldsymbol{\cdot}{\hat{\mathbf{e}}}^{\mathbf{2}} \right)$ is a random variable. The regression coefficient is b = cov(**X**, **Y**) / var(**X**), therefore cov(**X**, **Y**) = var(**X**). Therefore, Eq can be rewritten as

$\sigma_{d}^{2}= \sigma_{Y}^{2}-\sigma_{X}^{2}$ (Eq2)

When there is negligible G$\times$E interaction, bivariate GREML can be expressed as $y_{i}$ = $\alpha_{i0}$ + $e_{i}^{*}$ with $e_{i}^{*}\approx e_{i}$. Because MRNM is an expanded bivariate GREML, the values for $\hat{\sigma}_{e}^{2}$ can be similar, higher or lower than $\hat{\sigma}_{e*}^{2}$.

If $\hat{\sigma}_{e}^{2}$ is the same as $\hat{\sigma}_{e*}^{2}$ (i.e $e_{i}= e_{i}^{*}$), **X** and **Y** were not different and there is no sampling variance of the difference ($\sigma_{d}^{2}$ = 0). Also, it is possible that $\sigma_{X}^{2}$ is higher than $\sigma_{Y}^{2}$ due to a possible factor inflating $\sigma_{X}^{2}$ in MRNM. The estimated residual variance from MRNM can be written as $e_{i}= f+ e_{i}^{*}$. From MRNM, $\mathbf{X}= \hat{\sigma}_{e}^{2}=E[\left( {\hat{\mathbf{e}}}^{\boldsymbol{*}}+\hat{\mathbf{f}} \right)$^2^]$=E\left( {\hat{\mathbf{e}}}^{\mathbf{*2}} \right)+E\left( {\hat{\mathbf{f}}}^{\mathbf{2}} \right)-2E\left( {\hat{\mathbf{e}}}^{\mathbf{*}}\cdot\hat{\mathbf{f}} \right)$. From bivariate GREML, $\mathbf{Y}= \hat{\sigma}_{e^{*}}^{2}$. Hence, **X** can be expressed as a linear function of **Y** as **X** = **Y**b + $\lambda$ where b is a regression coefficient (b = 1) and $\lambda$ = $E\left( {\hat{\mathbf{f}}}^{\mathbf{2}} \right)-2E\left( {\hat{\mathbf{e}}}^{\mathbf{*}}\cdot\hat{\mathbf{f}} \right)$ = $E\left( {\hat{\mathbf{f}}}^{\mathbf{2}} \right)$ is a random variable. The regression coefficient for **X** is b = cov(**X**, **Y**) / var(**Y**), cov(**X**, **Y**) = var(**Y**). Eq1 can be rewritten as

$\sigma_{d}^{2}= \sigma_{X}^{2}-\sigma_{Y}^{2}$ (Eq3)

Considering Eq2 and Eq3 together, the sampling variance of the difference between bivariate GREML and MRNM can be generally expressed as

$$\sigma_{d}^{2}= {|\sigma}_{Y}^{2}-\sigma_{X}^{2}|$$
